# Non-Dioxin-Like Polychlorinated Biphenyls Inhibit G-Protein Coupled Receptor-Mediated Ca^2+^ Signaling by Blocking Store-Operated Ca^2+^ Entry

**DOI:** 10.1371/journal.pone.0150921

**Published:** 2016-03-10

**Authors:** Se-Young Choi, Keimin Lee, Yurim Park, Seung-Hyun Lee, Su-Hyun Jo, Sungkwon Chung, Kyong-Tai Kim

**Affiliations:** 1 Department of Physiology, Dental Research Institute, Seoul National University School of Dentistry, Seoul, Korea; 2 Department of Life Sciences, Division of Integrative Bioscience and Biotechnology, Pohang University of Science and Technology, Pohang, Korea; 3 Department of Physiology, Samsung Biomedical Research Institute, Sungkyunkwan University School of Medicine, Suwon, Korea; 4 Department of Physiology, Kangwon National University School of Medicine, Chuncheon, Korea; University of Debrecen, HUNGARY

## Abstract

Polychlorinated biphenyls (PCBs) are ubiquitous pollutants which accumulate in the food chain. Recently, several molecular mechanisms by which non-dioxin-like (NDL) PCBs mediate neurodevelopmental and neurobehavioral toxicity have been elucidated. However, although the G-protein coupled receptor (GPCR) is a significant target for neurobehavioral disturbance, our understanding of the effects of PCBs on GPCR signaling remains unclear. In this study, we investigated the effects of NDL-PCBs on GPCR-mediated Ca^2+^ signaling in PC12 cells. We found that *ortho*-substituted 2,2’,6-trichlorinated biphenyl (PCB19) caused a rapid decline in the Ca^2+^ signaling of bradykinin, a typical G_q_- and phospholipase Cβ-coupled GPCR, without any effect on its inositol 1,4,5-trisphosphate production. PCB19 reduced thapsigargin-induced sustained cytosolic Ca^2+^ levels, suggesting that PCB19 inhibits SOCE. The abilities of other NDL-PCBs to inhibit store-operated Ca^2+^ entry (SOCE) were also examined and found to be of similar potencies to that of PCB19. PCB19 also showed a manner equivalent to that of known SOCE inhibitors. PCB19-mediated SOCE inhibition was confirmed by demonstrating the ability of PCB19 to inhibit the SOCE current and thapsigargin-induced Mn^2+^ influx. These results imply that one of the molecular mechanism by which NDL-PCBs cause neurobehavioral disturbances involves NDL-PCB-mediated inhibition of SOCE, thereby interfering with GPCR-mediated Ca^2+^ signaling.

## Introduction

Polychlorinated biphenyls (PCBs) are some of the most ubiquitous environmental contaminants presently originating from industry. Their characteristics (e.g. inflammability and electric conductivity) have brought them to a range of applications such as coolants, flame retardants, and hydraulic fluids [1, 2]. Furthermore, the low water solubility and degradability of PCBs enable them to be readily deposited in human, fish, bird and plant tissues, thereby accumulating at several stages of the food chain [3]. This accumulation is problematic, because PCBs are toxic to nearly every tissue of humans and animals, including those of the endocrine, immune, metabolic, reproductive, and nervous systems [2]. Moreover, PCBs have been reported to be highly neurotoxic, with exposure to PCBs resulting in neurodevelopmental impairment and neurobehavioral disturbances [4–6]. Since PCBs tend to accumulate in blood and are secreted via breast milk [7–8], wide and intensive interest has also been given to the effects of PCBs on motor activity, learning, and memory attention in infants and children [9–12].

PCBs have been classified into dioxin-like (DL) and non-dioxin-like (NDL) groups [13]. DL-PCBs with chlorine atoms at the *meta* or *para* position of the phenyl ring show coplanar or dihedral molecular shape. On the other hand, NDL-PCBs are molecules containing chlorine substitutions at the *ortho* position of the phenyl ring and exhibiting a toxicological profile distinct from that of DL-PCBs [13]. NDL-PCBs have been shown to act via a pathway that is independent from the activation of the arylhydrocarbon receptor (AhR), a common mediator for the actions of DL-PCBs and 2,3,7,8-tetrachlorodibenzo-p- dioxin (TCDD). The most striking attribute of NDL-PCBs is their ability to modulate intracellular Ca^2+^ signaling. NDL-PCBs induce inositol phosphate accumulation [14], disrupt microsomal Ca^2+^ transport [15], change phospholipase A_2_ activity [16], and increase Ca^2+^ release from ryanodine receptors-sensitive intracellular Ca^2+^ pools [17–19]. NDL-PCBs-triggered sustained increase in cytosolic Ca^2+^ level thereby perturb Ca^2+^-triggered physiological responses and subsequent gene expression [20], and induce mitochondrial dysfunction [21].

However NDL-PCBs show more broad spectrum of neurotoxicity. NDL-PCBs increase a risk of autism spectrum disorder and/or attention deficit hyperactive disorder, which is hard to be simply explained with sustained Ca^2+^ increase and subsequent neuronal cell death [6]. Nonetheless, no information has been reported to date regarding potential crosstalk between NDL-PCBs and other neurotransmitters, especially with respect to G-protein coupled receptor (GPCR)-mediated Ca^2+^ signaling. Changes in neurotransmitter-mediated signaling are of great consequence, since they potentially affect neuronal cell-to-cell communication and can result in drastic neurophysiological perturbations. Because of their roles in hormonal and neurotransmitter function, GPCRs are especially critical targets for neurotoxic agents. Here, we report that NDL-PCBs block GPCR-mediated Ca^2+^ signaling pathways by inhibiting store-operated Ca^2+^ entry (SOCE). SOCE, also referred to as capacitative Ca^2+^ entry, comprises one of the key mechanisms by which GPCRs and phospholipase C (PLC) mediate increases in cytosolic Ca^2+^ levels. The aim of this study was to elucidate the cellular mechanisms by which NDL-PCBs perturb neuronal GPCR signaling.

## Results

### PCB19 inhibits bradykinin-induced Ca^2+^ signaling without any effect on phospholipase C activity

**Fig 1:**
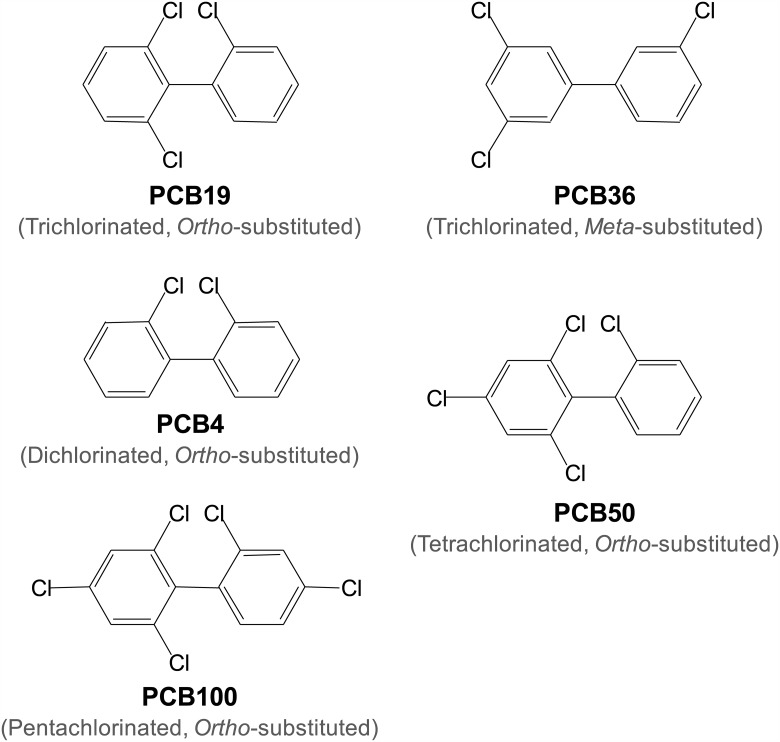
Structures of PCBs. PCB4 (2,2’-dichlorinated biphenyl), PCB19 (2,2’,6-trichlorinated biphenyl), PCB50 (2,2’,4,6-tetrachlorinated biphenyl), and PCB100 (2,2’,4,4’,6-pentachlorinated biphenyl) have chlorine atoms at the *ortho* position of the phenyl ring, whereas PCB36 (3,3’,5-trichlorinated biphenyl) contains chlorine substitution at the *meta* position of the phenyl ring.

**Fig 2:**
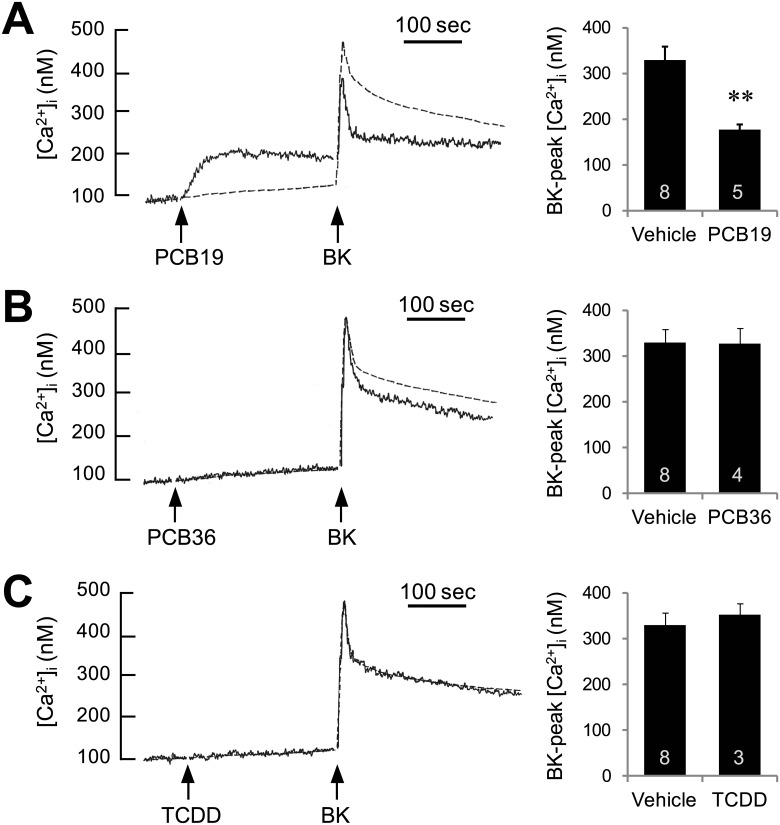
PCB19 inhibits bradykinin-induced increases of [Ca^2+^]_i_ in PC12 cells. (right) Fura-2-loaded PC12 cells were challenged with 50 μM PCB19 (**A**), 50 μM PCB36 (**B**), or 50 nM TCDD (**C**) and subsequently treated with 300 nM bradykinin. Responses to bradykinin alone, without PCB pretreatment, are also depicted (dotted traces). BK, bradykinin. (left) Peak changes in bradykinin-induced [Ca^2+^]i increase were quantitatively analyzed. Number of experiments are depicted in bar graph and each point represents mean ± SEM. **P < 0.01.

PC12 cells have classically been used to study the neurotoxicological properties of PCBs (Fig 1), as well as to characterize G-protein coupled receptors, for decades [22–24]. We examined the effect of PCB19 on GPCR-mediated [Ca^2+^]_i_ increases in PC12 cells, and confirmed the previous finding that 50 μM PCB19 induces a sustained increase in intracellular Ca^2+^ levels (Fig 2A). Interestingly, we also found that PCB19 partially inhibited bradykinin-induced [Ca^2+^]_i_ increases; furthermore, this inhibition was markedly enhanced in the “Ca^2+^-decreasing state” (*P* = 0.0038, t(11) = 3.653) (Fig 2A). These data suggest that PCB19 eventually weakens bradykinin receptor-mediated Ca^2+^ signaling. Moreover, neither PCB36 (AhR-activating DL-PCB) nor TCDD (AhR-activating dioxin) stimulated any Ca^2+^ increase by themselves, and both were also less effective than PCB19 to inhibit subsequent bradykinin-induced Ca^2+^ increase (Fig 2B and 2C).

**Fig 3:**
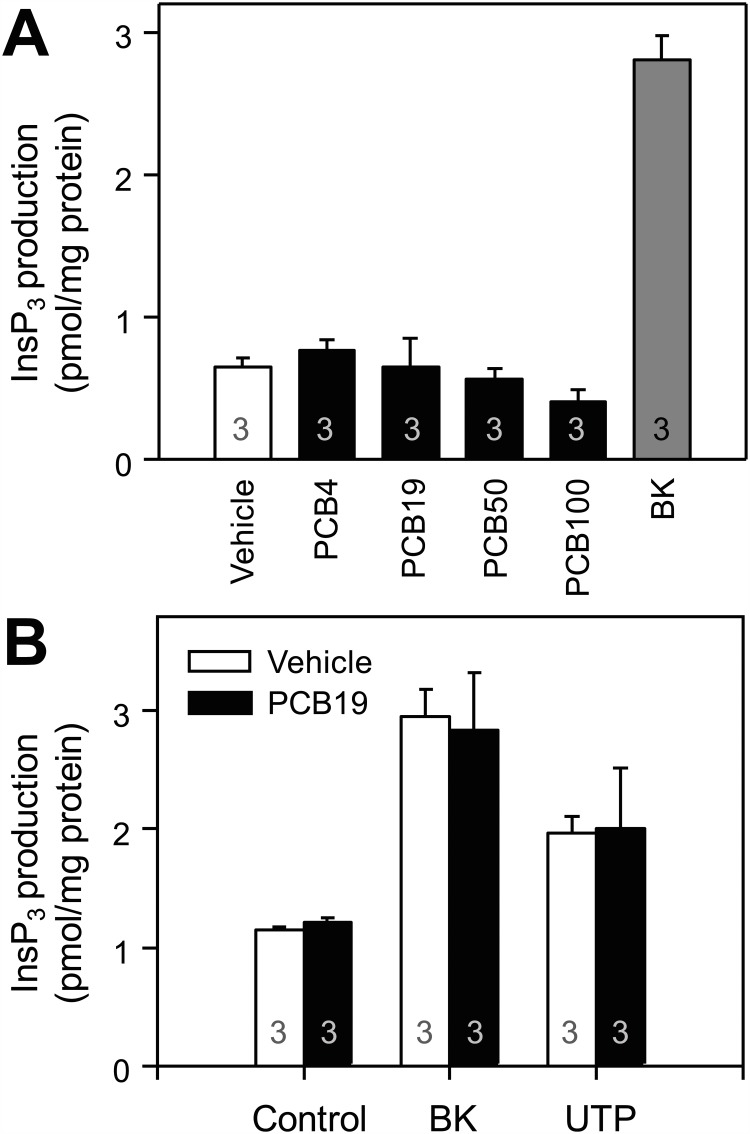
PCB19 does not affect InsP_3_ production. **A,** PC12 cells were treated with 50 μM PCB4, PCB19, PCB50, or PCB100, or 300 nM bradykinin, for 15 seconds. Subsequently, InsP_3_ production was measured. **B,** Cells were preincubated either with (black bar) or without (blank bar) 50 μM PCB19 for 3 min and then treated with 300 nM bradykinin for 15 seconds. Number of experiments are depicted in bar graph and each point represents mean ± SEM. BK, bradykinin.

Activation of GPCRs and PLC results in elevated [Ca^2+^]_i_ through a mechanism involving inositol 1,4,5-trisphosphate (InsP_3_)-dependent Ca^2+^ release from internal stores and subsequent SOCE from the extracellular space [25]. Thus, GPCR-mediated Ca^2+^ signaling is modulated at multiple levels, including the receptor itself, G-proteins, PLC, the InsP_3_ receptor, and the Ca^2+^ pool, as well as SOCE. To test whether PCB19 affects GPCR signaling, such as receptor activation and/or PLC activation, we examined whether NDL-PCBs affected InsP_3_ production. We found that NDL-PCBs, including PCB4 and PCB19, did not increase cytosolic InsP_3_ levels, whereas treatment with bradykinin successfully generated InsP_3_ (Fig 3A). Furthermore, NDL-PCBs did not inhibit either bradykinin- or UTP- (another Gq-coupled P2Y2 receptor agonist) induced InsP_3_ production (Fig 3B). Thus, we conclude that NDL-PCB-mediated inhibition of SOCE does not involve regulation of PLC activity.

### PCB19-induced Ca^2+^ influxes are relatively smaller than Ca^2+^ release

**Fig 4:**
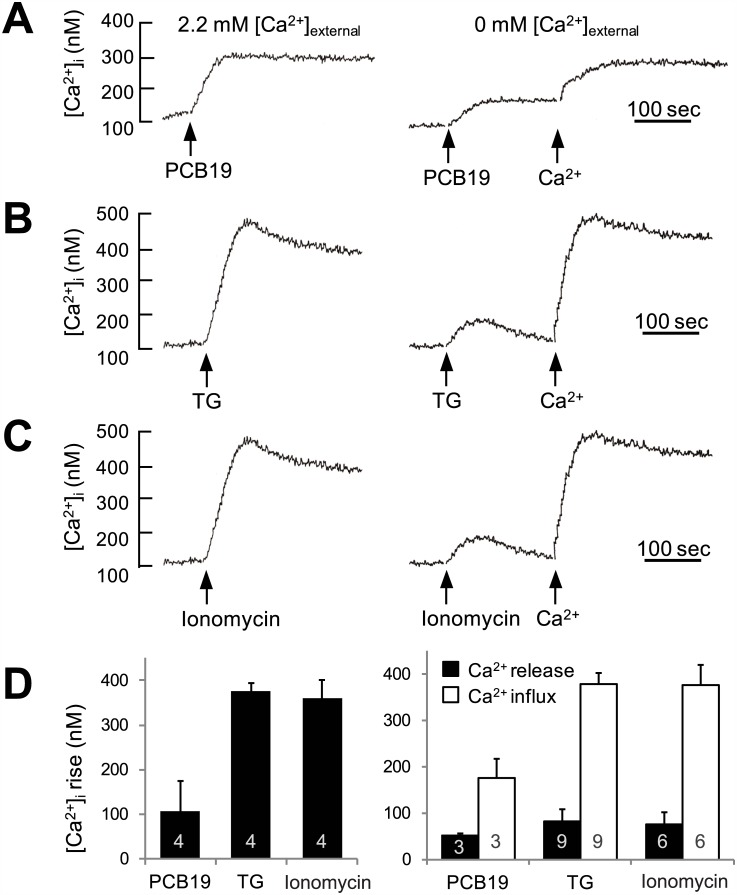
Ca^2+^ influxes stimulated by PCB19 are relatively small compared to those stimulated by intracellular Ca^2+^-mobilizing chemicals. **A**, Fura-2-loaded PC12 cells were challenged with 50 μM PCB19 in the presence (left) or absence (right) of 2.2 mM extracellular free Ca^2+^. Ca^2+^ increases were also monitored upon reintroduction of 2.2 mM CaCl_2_ in the condition lacking extracellular Ca^2+^. **B and C,** Experiments were performed as in (**A**), but with the addition of 1 μM thapsigargin **(B)** or 300 nM ionomycin **(C). D,** Peak height of [Ca^2+^]i increase was monitored and represented as mean ± SEM. **E and F,** Cells were treated with 300 nM ionomycin in the absence of extracellular free Ca^2+^ with (gray trance) or without (black trace) the pretreatment of 50 μM PCB19 for 100 sec. Ca^2+^ influx was then measured upon reintroduction of 2.2 mM CaCl_2_ (Ca^2+^) into the extracellular space to monitor the ionomycin-induced Ca^2+^ influx. Number of experiments are depicted in bar graph and each point represents mean ± SEM. TG, thapsigargin.

**Fig 5:**
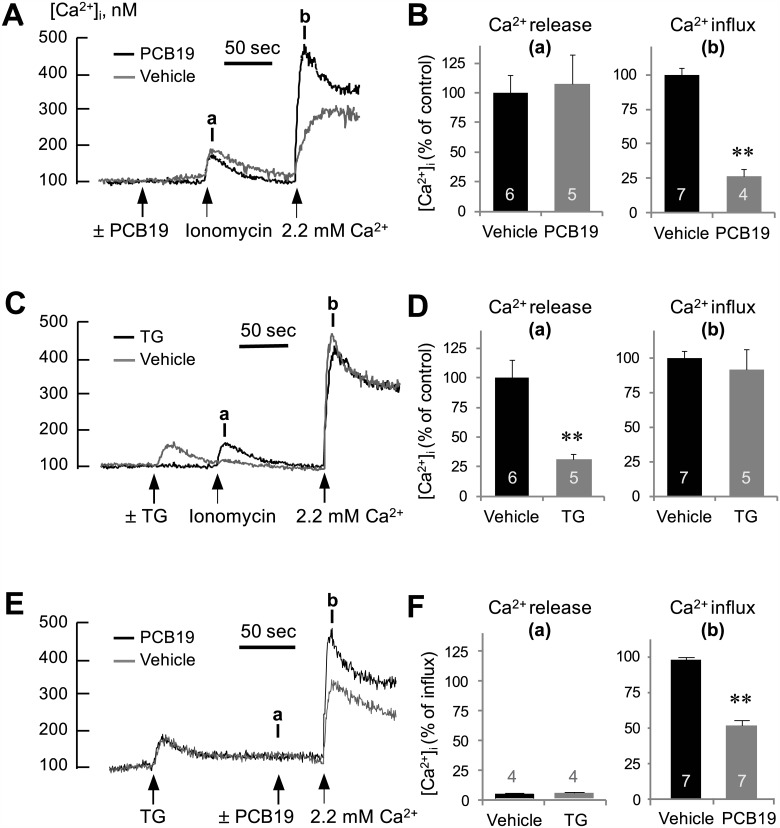
PCB19 inhibits ionomycin and thapsigargin-induced Ca^2+^ influxes. **(A, C, E)** Fura-2-loaded PC12 cells were incubated in Ca^2+^-free Locke’s solution, challenged with 50 μM PCB19, 1 μM thapsigargin or 300 nM ionomycin, and treated with 2.2 mM CaCl_2_ at the indicated time (arrow). **(B, D, F)** The [Ca^2+^]i level at point **a** (Ca^2+^ release) and **b** (Ca^2+^ influx) were quantitatively analyzed using calcium traces and expressed as % of controls. Number of experiments are depicted in bar graph and each point represents mean ± SEM. TG, thapsigargin. **P < 0.01.

Since the inhibitory effect of PCB19 on bradykinin-induced Ca^2+^ increase was more prominent in the decay phase than the initial phase, we focused subsequent studies on intracellular Ca^2+^ release and SOCE rather than on the earlier steps of GPCR and PLC activation. Similar to PCB19 (Fig 4A), thapsigargin (an inhibitor of the sarco/endoplasmic Ca^2+^ ATPase) depleted intracellular Ca^2+^ pools and induced subsequent SOCE (Fig 4B). However, the amplitude of PCB19-induced Ca^2+^ influx induced by reintroduced CaCl_2_ in the Ca^2+^-free condition was much smaller than that induced by thapsigargin-induced Ca^2+^ influx, even though the amplitude of PCB19-induced Ca^2+^ release in the Ca^2+^-free condition was similar to that induced by thapsigargin. This same trend was observed upon treatment with ionomycin, which is a Ca^2+^ ionophore and induces both Ca^2+^ release from intracellular stores, as well as Ca^2+^ influx from the extracellular space. Even though 300 nM ionomycin induced an amount of Ca^2+^ release similar to that induced by PCB19, the ionomycin-induced Ca^2+^ increase in the presence of extracellular Ca^2+^ was much larger than that induced by PCB19, most likely because less Ca^2+^ influx is triggered by PCB19 (Fig 4D). Also we found that thapsigargin or ionomycin evoked rapid and transient Ca^2+^ release, which returned to baseline within 1–2 min. By contrast, PCB19 induced a gradual and prolonged Ca^2+^ release, which did not return to basal level within the recording period. Considering the comparatively small Ca^2+^ influx triggered by PCB19, we hypothesized that PCB19 inhibits SOCE. In extracellular Ca^2+^ free condition, pretreatment with PCB19 did not affect ionomycin-induced Ca^2+^ release but blocked subsequent ionomycin-induced Ca^2+^ influx (*P* < 0.0001, t(9) = 9.233) (Fig 5A and 5B). The results are contrasted with another results that thapsigargin inhibits ionomycin-induced Ca^2+^ release but does not affect Ca^2+^ influx (*P* = 0.003, t(9) = 4.029) (Fig 5C and 5D) and confirmed the PCB19-induced SOCE inhibition. We also found that PCB19 successfully inhibited thapsigargin-induced Ca^2+^ influx in the experiment with PCB19 challenge before the reintroduction of 2.2 mM extracellular Ca^2+^ (*P* < 0.0001, t(12) = 10.42) (Fig 5E and 5F).

### NDL-PCBs inhibit ionomycin- or thapsigargin-induced [Ca^2+^]_i_ increases via SOCE

**Fig 6:**
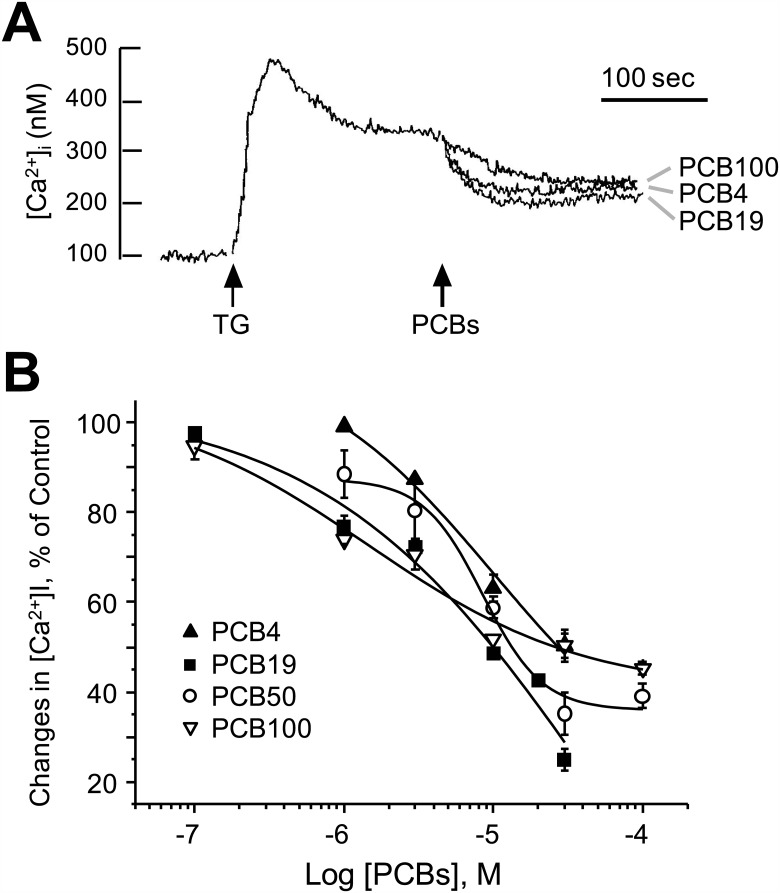
PCB19 blunts thapsigargin-induced increases in sustained [Ca^2+^]_i_ levels. **A**, Fura-2-loaded PC12 cells were treated with 1 μM thapsigargin; 5 minutes later (at the sustained phase), cells were challenged with 50 μM of either PCB4, PCB19, or PCB100. Data presented include typical Ca^2+^ traces from more than five independent experiments. **B**, Concentration-dependent effects of PCBs on thapsigargin-induced SOCE. Decreases in Ca^2+^ levels were monitored upon stimulation with various concentrations of PCB4 (filled triangles), PCB19 (filled squares), PCB50 (blank circles), and PCB100 (blank triangles). Net decreases in [Ca^2+^]_i_ are expressed as % of controls (thapsigargin-induced Ca^2+^ levels without PCB19 treatment). Each point shown was obtained from triplicate experiments and represents the mean ± SEM. TG, thapsigargin.

To test our hypothesis that PCB19 inhibits SOCE, we next devised a method of teasing apart Ca^2+^ release from Ca^2+^ influx, both of which are potentially affected by PCB19 treatment, in order to precisely define the effects of PCB19 on SOCE. Towards this end, we added PCB19 during the sustained Ca^2+^ phase evoked by thapsigargin, which itself depletes intracellular Ca^2+^ pools, thereby eliminating any PCB19-induced Ca^2+^ release. Thapsigargin treatment led to a prolonged high level of [Ca^2+^]i because of the interplay between Ca^2+^ release and SOCE. In this condition, PCB19 decreased thapsigargin-induced sustained Ca^2+^ levels (Fig 6A). Of particular note was our observation that PCB19 showed an inhibitory effect at the time point at which thapsigargin had already depleted Ca^2+^ pools and induced SOCE. Since these experimental conditions were able to suitably dissect the effect of PCB19 on SOCE alone, we proceeded to test the effects of other NDL-PCBs on SOCE. In addition to PCB19, PCB4, PCB50, and PCB100 also inhibited thapsigargin-evoked SOCE in concentration-dependent manners (Fig 6B). The potency order of NDL-PCBs with respect to SOCE inhibition was PCB19 = PCB100 ≥ PCB50 ≥ PCB4; however, these differences were rather subtle. While PCB19, PCB4, PCB50, and PCB100 showed complete inhibition of thapsigargin-evoked SOCE, PCB36 showed marginal inhibition than other NDL-PCBs, and TCDD did not affect SOCE at all (S1 Fig).

### PCB19 inhibits store-operated currents and Ca^2+^ influx

**Fig 7:**
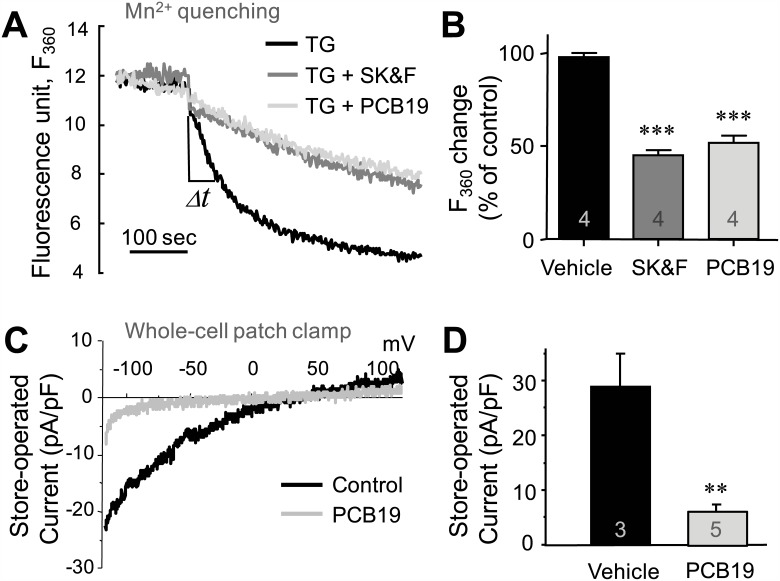
PCB19 inhibits store-operated cation entry. **A**, Mn^2+^-induced fura-2 fluorescence quenching was recorded in fura-2/AM-loaded PC12 cells. The fluorescence intensities at 360 nm (F_360_) was monitored with 1 mM MnCl_2_ (arrow), after the preincubation of thapsigargin (TG) with PCB19 or SK&F96365 (SK&F) in the absence of extracellular free Ca^2+^. **B**, The changes in time (Δt) during the fluorescence changes (arbitrary units) were quantitatively analyzed with the results in **A**. **C,** Ca^2+^ store depletion-induced cation influx was measured in PC12 cells with whole-cell patch clamp experiments. Currents were activated following dialysis with 10 mM BAPTA and ramp pulses of membrane potentials from -100 to +100 mV were applied to monitor SOCE current. Typical traces of Ca^2+^ store depletion-induced cation influxes with (gray trace) and without (black trace) 50 μM PCB19 are depicted. **D,** Comparison of average peak store-operated current densities (pA/pF). Number of experiments are depicted in bar graph and each point represents mean ± SEM. **P < 0.01.

Although we excluded the possibilities that PCB19 affects GPCR or PLC activities, the possibility still remains that PCB19 affects Ca^2+^ signaling machinery to replenish Ca^2+^ stores and/or to recover [Ca^2+^]i levels to the basal level more efficiently (for example, by activating plasma membrane Ca^2+^-ATPases (PMCA) [26] or Na^+^/Ca^2+^ exchangers (NCX) [27]). In this scenario, PCB19 could inhibit SOCE indirectly, without having any direct effect on SOCE-mediated Ca^2+^ influx. To test the possibility of an indirect SOCE inhibition via a fast recovery of [Ca^2+^]i levels, we used bepridil (Na^+^ site specific NCX inhibitor), and caloxin (isoform-nonselective PMCA inhibitor). Interestingly PCB19-mediated SOCE inhibition is not affected by the presence of bepridil or caloxin, implying that PCB19’s effect is not contributed by the activation of PMCA or NCX (S2 Fig). Moreover, to address a direct effect on SOCE-mediated Ca^2+^ influx, we monitored SOCE-induced Mn^2+^ quenching under the presence of PCB19. When fura-2 is excited at 360 nm (its isosbestic wavelength), its fluorescence is not affected by cytosolic Ca^2+^, whether originating from the intracellular pool or the extracellular space. Therefore, if Mn^2+^ is added in the extracellular solution, any changes in fluorescence result from binding of fura-2 to Mn^2+^, the latter of which is only able to enter cells from extracellular space. We found that PCB19 decreased the rate of thapsigargin-induced fluorescence quenching, indicating that thapsigargin-induced SOCE is specifically inhibited by PCB19 like as a SOCE inhibitor 1-{-[3-(4-methoxyphenyl)propoxy]-4-methoxyphenyl}-1H- imidazole hydrochloride (SK&F96365) [28] (vehicle vs SK&F96365: *P* < 0.0001, t(7) = 20.01; vehicle vs PCB19: *P* < 0.0001, t(10) = 9.427) (Fig 7A and 7B). We also confirmed the effect of PCB19 by measuring store-operated currents directly by whole-cell patch clamping experiments. The infusion of BAPTA, an intracellular Ca^2+^ chelator, via a patch pipette in the whole cell configuration triggers store-operated currents. Also thapsigargin and InsP_3_ were included in pipette solution to deplete the intracellular calcium stores. Interestingly, store-operated currents were completely eliminated by preincubation with PCB19 (*P* = 0.0016, t(6) = 5.439) (Fig 7C and 7D), suggesting that PCB19 directly targets channels involved in store-operated currents rather than Ca^2+^ signaling machinery.

### PCB19 inhibits SOCE in a similar manner of known SOCE antagonists

**Fig 8:**
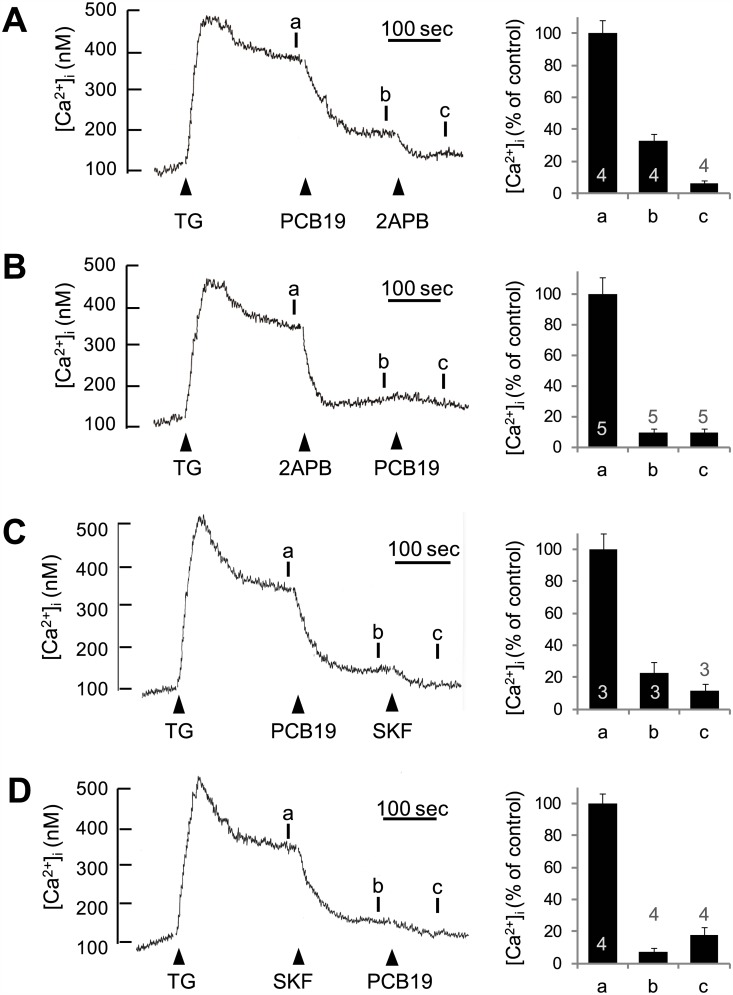
PCB19 inhibits thapsigargin-induced SOCE in a manner similar to other SOCE antagonists. **A,** Fura-2-loaded PC12 cells were treated with 1 μM thapsigargin (TG), then sequentially challenged with 100 μM PCB19 and 30 μM 2-aminoethylphenyl borate (2APB). **B,** Cells were treated with 1 μM thapsigargin, challenged with 20 μM 2APB, and then treated with 50 μM PCB19. **C,** Cells were treated with 1 μM thapsigargin, then challenged sequentially with 100 μM PCB19 and 20 μM SK&F96365 (SKF). **D,** Cells were treated with 1 μM thapsigargin, challenged with 20 μM SK&F96365, and then treated with 50 μM PCB19. The [Ca^2+^]i level at point **a**, **b**, and **c** were quantitatively analyzed using calcium traces. Number of experiments are depicted in bar graph and each point represents mean ± SEM.

Finally, we compared PCB19-mediated inhibition of SOCE to that of previously reported SOCE-modulating chemicals including SK&F96365 and 2-aminoethyldiphenyl borate (2APB) [29]. 2APB decreased thapsigargin-induced sustained elevations of Ca^2+^ levels in a manner similar to PCB19; furthermore, subsequent addition of PCB19 did not increase the amplitude of this inhibition (Fig 8A), implying that PCB19 and 2APB act either on the same target or on parallel targets in the common pathway. This conclusion was confirmed by the result that 2APB with different concentrations (10–100 μM) commonly show the overlapped effect of 2APB and PCB19 (S3 Fig). The same experiment generated similar results when performed in the reverse order (e.g. PCB19 first, then 2APB; Fig 8B) and when performed with the other SOCE inhibitor, SK&F96365 (Fig 8C and 8D). Together, these results suggest that PCB19, SK&F96365, and 2APB share a common target by which they inhibit SOCE.

## Discussion

GPCR-mediated Ca^2+^ signaling is known to be modulated by multiple mechanisms. Activation of GPCRs and PLC produces InsP_3_, which binds to InsP_3_ receptors near intracellular Ca^2+^ stores (e.g. endoplasmic reticulum (ER)) and induces Ca^2+^ release from these stores, thereby depleting them of Ca^2+^. After Ca^2+^ is depleted from intracellular stores, they must be replenished. This occurs via Ca^2+^ influx from the extracellular space in a process known as SOCE [25,30]. Therefore, SOCE serves a very important role in enabling GPCR-mediated Ca^2+^ signaling. In this study, we demonstrated for the first time that PCB19 inhibits SOCE, a process that is normally activated downstream from PLC activation and depletion of thapsigargin-sensitive Ca^2+^ stores. We present the following evidence in support of this idea: (1) PCB19 reduces bradykinin-induced Ca^2+^ increases without inhibiting bradykinin-induced InsP_3_ production, (2) PCB19 inhibits thapsigargin-induced Ca^2+^ influx as well as elevation of sustained Ca^2+^ levels, (3) PCB19 reduces store-operated currents triggered by intracellular challenges with Ca^2+^ chelators, and (4) PCB19 does not affect Ca^2+^ levels in cells treated with SOCE inhibitors.

It has been reported that NDL-PCBs themselves are capable of triggering Ca^2+^ release [31]. Numerous studies in the last decade have elucidated that NDL-PCBs induce Ca^2+^ release and its source is ryanodine receptor-Ca^2+^ channel complex type 1 (RyR1). Eversince Wong and Pessah (1996) firstly reported the NDL-PCB-mediated Ca^2+^ release [17], the structure-activity relationship of NDL-PCB on RyR1 activation has come to be found [19]. However, we found for the first time the NDL-PCB-induced SOCE inhibition which has been obscured by NDL-PCB-induced Ca^2+^. Thus, we conclude that PCB19 appears to have a dual effect on SOCE: (1) PCB19 activates SOCE by promoting Ca^2+^ release, but (2) PCB19 inhibits SOCE by blocking Ca^2+^ influx from the extracellular space.

Examining structure-activity relationships is a helpful approach for distinguishing target mechanisms when the chemical bears multiple target sites. In our study, we compared the effects of PCB4, PCB19, PCB50, and PCB100, and found that their inhibitory potencies against SOCE ranked in the following order: PCB19 = PCB100 ≥ PCB50 ≥ PCB4. Even though PCB36 and PCB19 are both trichlorinated biphenyls, they exhibited a large discrepancy in activities because of the substituted chlorine atom in the *ortho* position in PCB19. In addition, a PCB with five substituted chlorine atoms (i.e. PCB100) showed similar potency to those of PCBs with three or four substituted chlorine atoms (i.e. PCB19, PCB50). Also, we did not observe any significant differences in potencies between PCBs with (i.e. PCB50, PCB100) and without (i.e. PCB4, PCB19) a substituted chlorine atom in the *para* position. From these results we conclude that (1) only NDL-PCBs inhibit SOCE, whereas DL-PCBs are less effective on SOCE inhibition, and (2) neither the number nor the position of the chlorine atom substitution in NDL-PCBs correlates with the SOCE inhibition activity of the molecule. Comparing our data to previously reported studies of PCBs raises some interesting issues. First, NDL-PCBs have been reported to potentiate GABA_A_ channel activity, but the potency of this potentiation has been shown to decrease according to the number of the substituted chlorine atom, with *para*-substituted NDL having almost negligible potency [32]. Second, structure-activity relationship of SOCE inhibition is different from the one reported for ryanodine receptors, another target for NDL-PCBs [18–19], because *para*-substituted NDL was found to be active in SOCE inhibition with a strikingly different potency profile. Although complete structure-activity relationships require more data from additional PCBs to be fully constructed, we conclude that PCB-mediated inhibition of SOCE occurs through a unique mechanism which has not yet been described (e.g. independent of activation of either the ryanodine receptor or the GABA_A_ receptor).

PCB19 has been suggested to perturb membrane structure and affect channel activity. PCBs affect channels and ionotropic receptors, including the voltage-sensitive Ca^2+^ channel [23,24,33], the GABA_A_ receptor [32,34], and the hERG K^+^ channel [35]. Others have suggested an alternative possibility that lipid-soluble PCB19 can diffuse into the cell and affect cytosolic molecules, including machineries that control intracellular Ca^2+^ concentration. We think the activity profile of PCB19 in two different cells provides insights into the action mechanism. Recent investigations have revealed that the molecular players of SOCE are ORAI1 and STIM1 [36]. After Ca^2+^ pool depletion is recognized by STIM1 (an EF-hand containing Ca^2+^ sensor in ER), STIM1 moves to ER-plasma membrane junctions and opens ORAI1 channel by ORAI1-STIM1 interaction [36]. Variations in ORAI family (i.e. ORAI1α, ORAI1β, ORAI2, ORAI3) and STIM family (STIM1, STIM1L, STIM2.1, STIM2.2, STIM2.3) allows different characteristics of SOCE in different cell types [37]. Especially STIM1-mediated SOCE is reported in PC12 cells [38]. Further investigation such as subtype specific knock down approaches, visualization of SOCE components and structural analysis of co-crystals of PCBs will provide a much clearer view of the action mechanism of PCB.

In toxicological aspect, an important finding of this study was that NDL-PCBs affect GPCR signaling by inhibiting SOCE. Communication between neurons and other cells, a process heavily mediated by neurotransmitters, is known to be critical for neurons to fulfil their roles in higher brain functions. Therefore, GPCRs, which serve as receptors for neurotransmitters, are often effective targets for neurotoxicological agents. To date, PCB-induced GPCR modulation has been studied only on the level of receptor expression. Long-term exposure to PCB for the embryo and/or neonate causes decreases in muscarinic receptor expression [39,40]. Whereas these previous findings support the hypothesis that the neurodevelopmental toxicity of PCB is related to long-term changes in GPCR expression patterns, our findings explain the neurophysiological toxicity of PCB on a shorter time scale. Compounds that interfere with GPCR signaling can affect the efficiency of medicines known to modulate GPCR activity or alter the concentration of neurotransmitters needed to achieve a desired effect (for example, serotonin-specific reuptake inhibitors or monoamine oxidase inhibitors, used for treating major depression). Furthermore, they evoke neurobehavioral disturbances by causing changes in synaptic plasticity, such as affecting long-term potentiation or long-term depression. A large body of evidence has accumulated in support of the idea that the neurobehavioral toxicities of PCBs are due to their effects on synaptic plasticity [41–43]. Notably, GPCR signaling pathways are key modulators of synaptic plasticity and are crucial for evoking metabotropic glutamate receptor-dependent long-term depression [44] and for inducing experience-dependent synaptic modifications [45]. Importantly, this study identifies SOCE as a novel toxicological target of NDL-PCBs, thereby making an important contribution to our understanding of the mechanism of neurobehavioral toxicity of NDL-PCBs.

## Materials and Methods

### Chemicals

PCB4, PCB19, PCB36, PCB50, and PCB100 were obtained from Chem Service (West Chester, PA, USA). ATP, bradykinin, GTP, sodium methane sulfonate, glucose, N-hydroxyethylethylenediamine triacetic acid, 1,2-bis(2-aminophenoxy)ethane-N,N,N’,N’- tetraacetic acid (BAPTA), HEPES, and sulfinpyrazone were purchased from Sigma (St. Louis, MO, USA). SK&F96365 and 2-aminoethoxyphenyl borate (2-APB) were obtained from Tocris (Bristol, UK). Thapsigargin was purchased from Alomone Labs (Jerusalem, Israel). Fura-2/acetoxymethylester (Fura-2/AM) was obtained from Molecular Probes (Eugene, OR, USA). [^3^H] Inositol 1,4,5-trisphosphate (InsP_3_) was purchased from GE Healthcare Life Sciences (Amersham, Piscataway, NJ, USA). Bovine calf serum and horse serum were obtained from HyClone (Logan, UT, USA). RPMI 1640 and penicillin/streptomycin were purchased from Gibco (Grand Island, NY, USA).

### Cell culture

PC12 rat pheochromocytoma cells [46] were grown in RPMI 1640 medium supplemented with 10% (v/v) heat-inactivated bovine calf serum, 5% (v/v) heat-inactivated horse serum, and 1% (v/v) penicillin/streptomycin. The final concentrations of penicillin and streptomycin were 5 units/ml and 50 μg/ml, respectively. Culture medium was changed daily, and cells were subcultured weekly.

### Measurement of intracellular Ca^2+^ concentrations ([Ca^2+^]_i_)

The fluorescent Ca^2+^ indicator, fura-2, was used to determine [Ca^2+^]_i_ according to previously reported methods [47]. Briefly, cell suspensions were incubated in Locke's solution (154 mM NaCl, 5.6 mM KCl, 5.6 mM glucose, 2 mM CaCl_2_, 1.2 mM MgCl_2_, and 5 mM HEPES buffer adjusted to pH 7.4) supplemented with 3 μM fura-2/AM for 50 min at 37°C with continuous stirring. Loaded cells were then washed twice with Locke's solution; sulfinpyrazone (250 μM) was added to all solutions to prevent dye leakage. Fluorescence ratios were monitored using dual excitation wavelengths of 340 and 380 nm and detecting the ratio of resultant intensities at an emission wavelength of 500 nm. For the experiments in the extracellular Ca^2+^-free condition, cells were incubated with Ca^2+^-free Locke’s solution (156.2 mM NaCl, 5.6 mM KCl, 5.6 mM glucose, 1.2 mM MgCl_2_, 100 μM EGTA, 5 mM HEPES buffer adjusted to pH 7.4) for 30 sec (Fig 4) or 5 min (Fig 5), and then 2.2 mM CaCl_2_ was challenged to induce Ca^2+^ influx. Conversion of fluorescence ratios into [Ca^2+^]_i_ was performed as described [48].

### Measurement of InsP_3_ production

InsP_3_ mobilization was determined by competition assays using [^3^H]InsP_3_ as described previously [49,50]. Briefly, to quantify InsP_3_ production, confluent cells in 6-well plates were stimulated with the drugs of interest, followed by the addition of ice-cold 5% trichloroacetic acid containing 10 mM EGTA to terminate the reactions and lyse the cells. Lysate supernatants were saved, and trichloroacetic acid was extracted with diethylether. Aqueous fractions remaining after the final extraction were neutralized with 200 mM Trizma base adjusted to pH 7.4. 20 μl of the extract were added to 20 μl of assay buffer (0.1 M Tris buffer containing 4 mM EDTA) and 20 μl of [^3^H]InsP_3_ (100 nCi/ml). The resultant mixture was incubated for 15 min on ice and then centrifuged at 2,000 x *g* for 10 min. 100 μl of water and 1 ml of liquid scintillation cocktail were added to the pellet to measure its radioactivity. The InsP_3_ concentrations of the samples were determined by comparison to a standard curve and expressed as pmol/mg of protein. Total cellular protein concentrations were determined with the Bradford method after sonication of cells.

### Measurement of Ca^2+^ influx from extracellular space

The Mn^2+^ quenching assay was performed as previously described [51–52] to measure Ca^2+^ influx from the extracellular space. Briefly, fura-2-loaded cells were pretreated with thapsigargin, SK&F96364 and/or PCB19 in extracellular Ca^2+^-free condition. The slope of the Mn^2+^-induced changes in fluorescence intensities were monitored (wavelengths: 360 nm excitation and 510 nm emission) upon 1 mM MnCl_2_ treatment.

### Electrophysiology

Patch-clamp experiments were conducted in the whole-cell configuration using fire-polished pipettes with a final resistance of 3–5 MΩ. To establish the whole-cell configuration, cell-attached patches were generated, and the cell membrane underneath the patch pipette was ruptured by gentle suction. Store-operated currents were recorded as described previously [53] with the bath solution which had the following composition: 135 mM sodium methanesulfonate, 5 mM NaCl, 10 mM HEPES, and 10 mM N-hydroxyethylethylenediamine triacetic acid; pH was adjusted to 7.2 with HCl. The pipette solution had the following composition: 135 mM Cesium methanesulfonate, 10 mM BAPTA, 10 mM Hepes, 5 mM MgCl_2_ (pH 7.2 with CsOH). Thapsigargin (1 μM) and InsP_3_ (1 μM) were also included in pipette solution to deplete the intracellular calcium stores. To monitor SOCE current, cells at a holding potential of -70 mV were applied by ramp pulses of membrane potentials from -100 to +100 mV. Transient and leak currents were not canceled. Currents were sampled at 5 kHz and digitally filtered at 1 kHz using either an Axopatch 200B with a Digidata 1200 interface or a Multiclamp 700B amplifier with a Digidata 1440 interface. Data acquisition and analysis were performed using the pClamp program (Molecular Devices, Union City, CA, USA). All experiments were performed at room temperature (20–23°C).

### Data analysis

All quantitative data are expressed as means ± SEM. The Origin for Windows program (Microcal Software Inc., Northhampton, MA, USA) was used to calculate IC_50_ values. Differences were determined by one-way ANOVA and considered to be significant only for *P* values < 0.05.

## Supporting Information

S1 Fig TCDD and PCB36 do not affect thapsigargin-induced increases of [Ca^2+^]_i_. **A-B,** Fura-2-loaded PC12 cells were challenged with 50 nM TCDD (**A**), or 50 μM PCB36 (**B)** and subsequently treated with 1 μM thapsigargin (TG). **C-D,** Fura-2-loaded PC12 cells were treated with 1 μM thapsigargin (TG), then sequentially challenged with 50 nM TCDD (**C**), or 50 μM PCB36 (**D**). Responses to thapsigargin alone, without chemical pretreatment, are also depicted (dotted traces).
(TIF)

S2 Fig PMCA inhibitors fail to block the PCB19-mediated SOCE inhibition. **A,** Fura-2-loaded PC12 cells were treated with 1 μM thapsigargin (TG) in the absence (black trace) or the presence of 10 μM bepridil (dark gray trace) or 10 μM caloxin (light gray trace), and then treated with 50 μM PCB19. **B,** Net decreases in [Ca^2+^]_i_ are expressed as % of controls (thapsigargin-induced Ca^2+^ levels without PCB19 treatment). Number of experiments are depicted in bar graph and each point represents mean ± SEM.
(TIF)

S3 Fig PCB19 inhibits thapsigargin-induced SOCE in manner similar to 2APB. **A,** Fura-2-loaded PC12 cells were treated with 1 μM thapsigargin (TG), then sequentially challenged with indicated concentration of 2APB (10 μM, black trace; 20 μM, dark gray trace; 100 μM, light gray trace), and then treated with 50 μM PCB19. **B,** The [Ca^2+^]i level at point **a**, **b**, and **c** were quantitatively analyzed using calcium traces. Number of experiments are depicted in bar graph and each point represents mean ± SEM.
(TIF)
