# Supplementary figures and images for: Non-Dioxin-Like Polychlorinated Biphenyls Inhibit G-Protein Coupled Receptor-Mediated Ca2+ Signaling by Blocking Store-Operated Ca2+ Entry
Source: PLoS One. 2016 Mar 10;11(3):e0150921. doi: 10.1371/journal.pone.0150921 (PMC4786281; doi:10.1371/journal.pone.0150921)

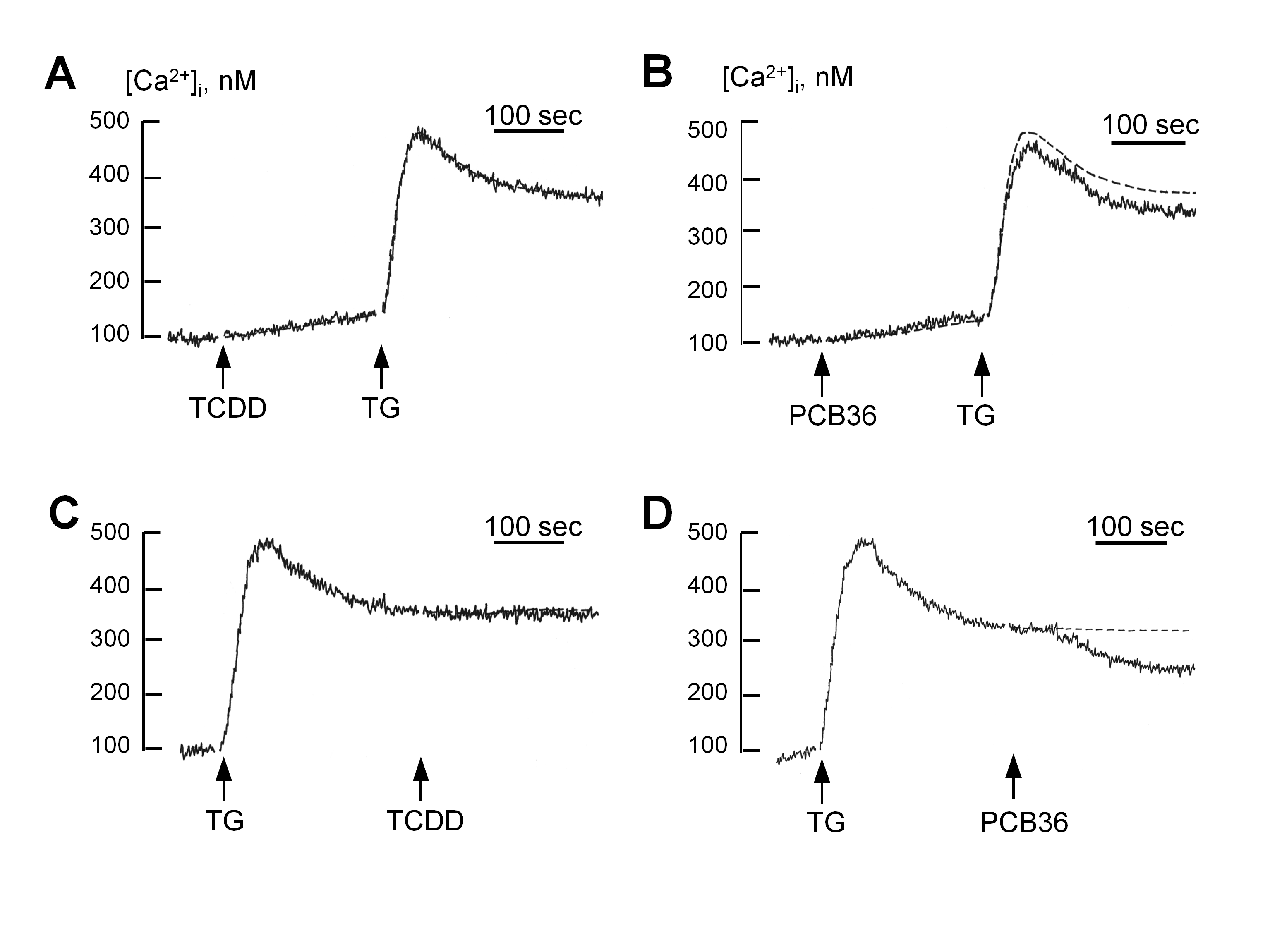

Supplement: S1 Fig — A-B, Fura-2-loaded PC12 cells were challenged with 50 nM TCDD (A), or 50 μM PCB36 (B) and subsequently treated with 1 μM thapsigargin (TG). C-D, Fura-2-loaded PC12 cells were treated with 1 μM thapsigargin (TG), then sequentially challenged with 50 nM TCDD (C), or 50 μM PCB36 (D). Responses to thapsigargin alone, without chemical pretreatment, are also depicted (dotted traces). (TIF) [file pone.0150921.s001.TIF]

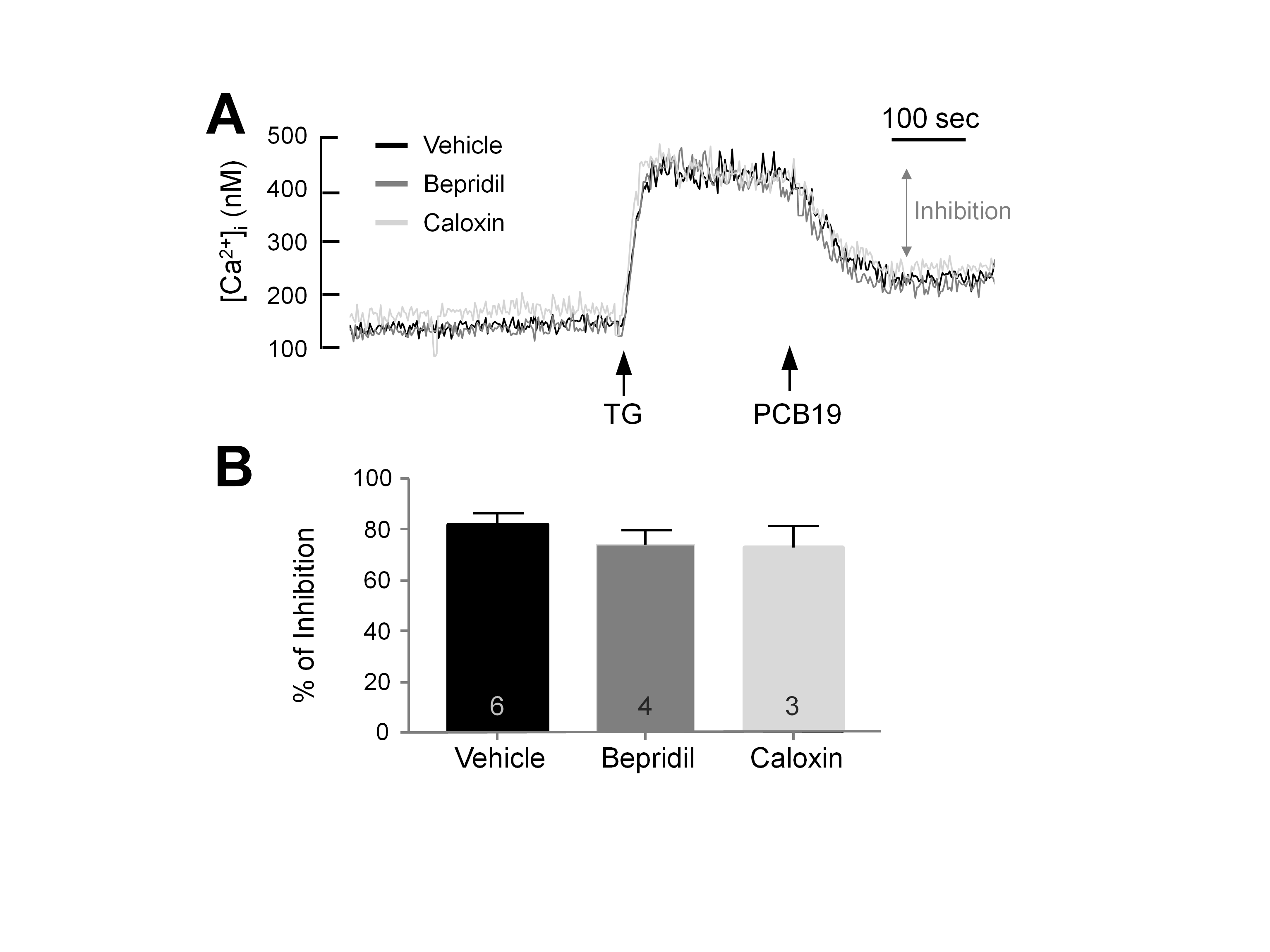

Supplement: S2 Fig — A, Fura-2-loaded PC12 cells were treated with 1 μM thapsigargin (TG) in the absence (black trace) or the presence of 10 μM bepridil (dark gray trace) or 10 μM caloxin (light gray trace), and then treated with 50 μM PCB19. B, Net decreases in [Ca2+]i are expressed as % of controls (thapsigargin-induced Ca2+ levels without PCB19 treatment). Number of experiments are depicted in bar graph and each point represents mean ± SEM. (TIF) [file pone.0150921.s002.TIF]

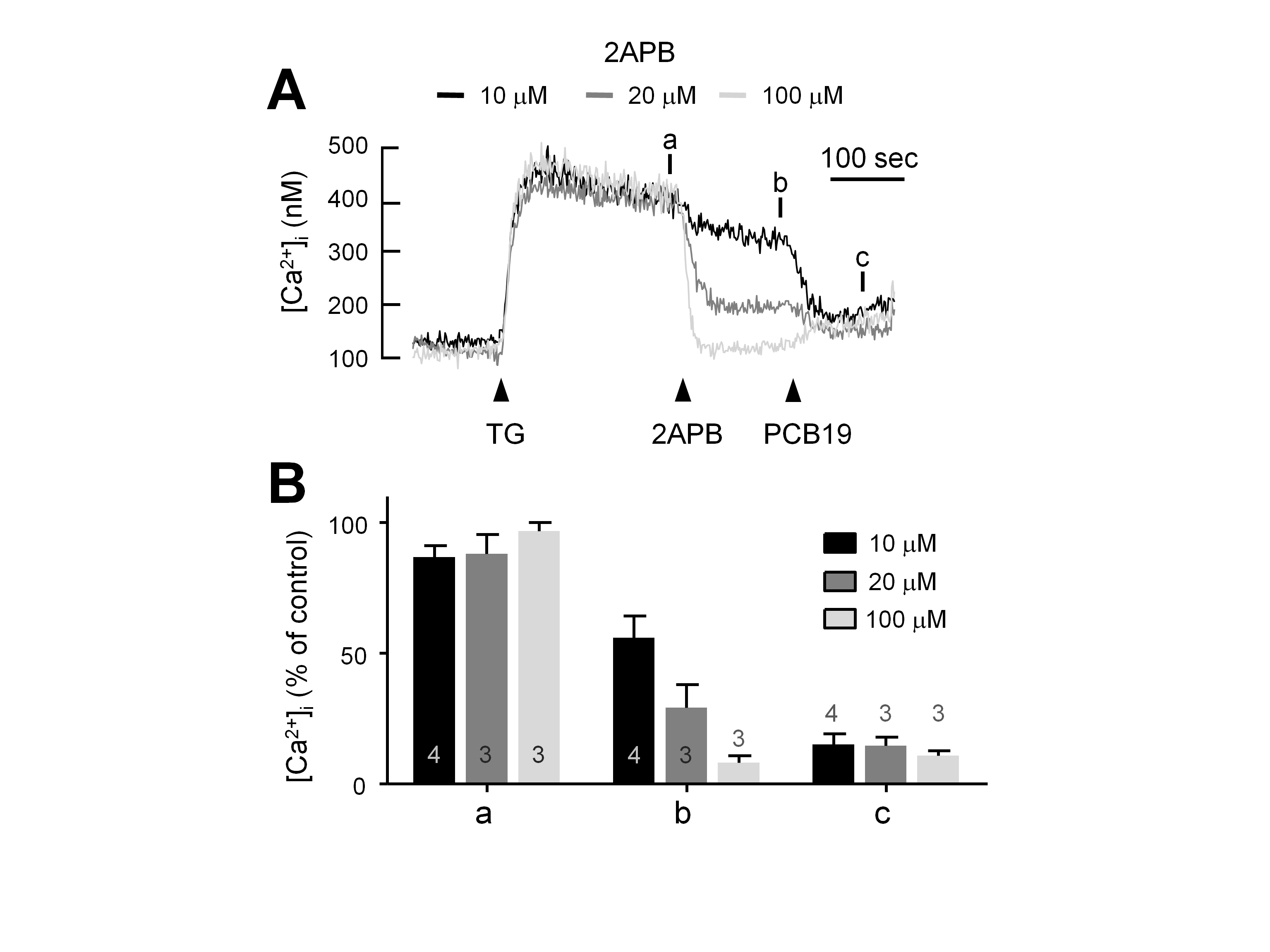

Supplement: S3 Fig — A, Fura-2-loaded PC12 cells were treated with 1 μM thapsigargin (TG), then sequentially challenged with indicated concentration of 2APB (10 μM, black trace; 20 μM, dark gray trace; 100 μM, light gray trace), and then treated with 50 μM PCB19. B, The [Ca2+]i level at point a, b, and c were quantitatively analyzed using calcium traces. Number of experiments are depicted in bar graph and each point represents mean ± SEM. (TIF) [file pone.0150921.s003.TIF]
